# The dsRNA-dependent kinase (PKR) inhibits the growth of *Leishmania major* via NF-κB-mediated genes

**DOI:** 10.1017/S0031182025100218

**Published:** 2025-07

**Authors:** Áislan de Carvalho Vivarini, Bianca Cristina Duarte Vivarini, Yuri Nunes Oliveira, Flavia Thiebaut, Evelize Folly das Chagas

**Affiliations:** 1 Department of Cellular and Molecular Biology, Fluminense Federal University, Niteroi, Rio de Janeiro, Brazil; 2 Department of Specialized and General Surgery, Fluminense Federal University, Niteroi, Rio de Janeiro, Brazil

**Keywords:** *Leishmania major*, macrophage, NF-κB, PKR

## Abstract

Parasites of the *Leishmania* species have been observed to infect macrophages and thereby modulate the host microbicidal responses, resulting in a wide spectrum of diseases. A comprehensive experimental mapping of the relationship between the double-stranded RNA protein kinase R (PKR) and NF-κB pathways in the outcome of the infection was conducted in an effort to improve the understanding of the biology associated with the parasites–host cell interaction. The results showed that in the absence of PKR and Type I Interferon (IFN) signalling, *L. major* infection was enhanced. The levels of PKR and gene promoter activation were evaluated. The results showed that infection did not induce PKR expression by inhibiting the phosphorylation of STAT1 and subsequent binding in the PKR promoter. However, infection induced PKR phosphorylation but did not prevent subsequent signalling through this pathway. To address the role of activation of these signalling, the induction of PKR-dependent gene expression was examined. Activation of the classical p65/p50 dimer was found to be dependent on the PKR in the *L. major* infection, which was essential for the induction of iNOS, IFNβ and tumour necrosis factor expression. In addition, macrophages treated with nuclear factor-kB inhibitors were more susceptible to infection. Furthermore, translocation of the p65/p50 to the promoters of these genes increased in a PKR-dependent manner. Collectively, these results suggest that macrophages retain their ability to induce important downstream effectors in PKR signalling. These effectors contribute to protection in pathogenesis, reducing parasite proliferation and regulating the inflammatory genes that, consequently, modulate the activation state of macrophages during infection.

## Graphical abstract

**Figure:**
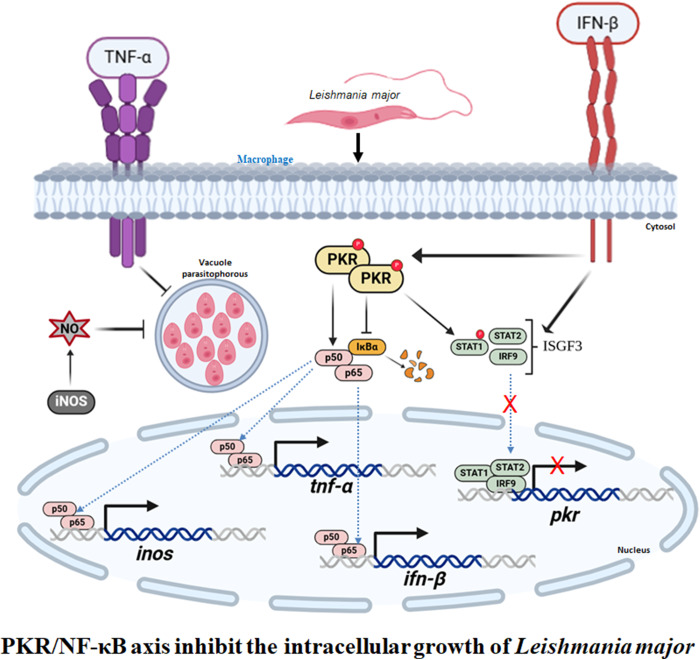

## Introduction

Human leishmaniasis is a well-studied model of an emerging disease caused by several species of protozoan parasites of the genus *Leishmania*. It is known to affect approximately 12 million people on all five continents (WHO, 2023). Once inside the host macrophage, *Leishmania* assumes the amastigote form (de Almeida et al., 2003) and depending on the species and the immunological status of the host, leishmaniasis presents with different clinical forms, ranging from cutaneous, mucocutaneous and diffuse cutaneous to visceral infections (McMahon-Pratt and Alexander, 2004; Schriefer et al., 2008). The immunological response of the host macrophages involves the induction of several genes that promote inflammation and pathogen resistance. However, *Leishmania* parasites have developed complex adaptive strategies and sophisticated mechanisms that allowed them to evade normal functions of macrophages and modulate host signalling pathways (Gregory and Olivier, 2005).

*Leishmania major*, a species predominantly found in the Middle East, Northern Africa and selected regions of China and India, was identified as the causative agent of cutaneous leishmaniasis. This disease manifests in the form of a solitary ulcerated lesion or disseminated cutaneous infiltrated plaques (Silveira et al., 2004, 2009; Gramiccia and Gradoni, 2005). Recent evidence highlighted some aspects in the immune response to *L. amazonensis* compared to *L. major* infections. In T cells derived from *L. amazonensis*-infected mice, in contrast to *L. major*, the presence of a type-1 or type-1/type-2 mixed phenotype was evident, in contrast to the response observed in *L. major* infections (Ji et al., 2005). In addition, studies showed that, unlike L. *major* infections, *L. amazonensis* modulated the early production of inflammatory cytokines and other immune molecules, including numerous central kinases (Ji et al., 2003).

Double-stranded-RNA (dsRNA)-dependent protein kinase R (PKR) was identified as a key component of the innate immune response, particularly during, viral infections due to its activation by viral dsRNA intermediates (Williams, 1999). Subsequently, PKR was shown to inhibit the protein synthesis by phosphorylating the eukaryotic translation initiation factor eIF2-α, its primary downstream substrate (Chong et al., 1992; Williams, 2001). In addition to its function as an antiviral response, PKR was implicated in a variety of biological processes, including apoptosis, transcription factors activation and lipid metabolism (Balachandran et al., 2000; Nakamura et al., 2010). Also noteworthy is its recently described role in inflammasome activation and actin dynamics (Irving et al., 2012). PKR can be activated by a number of other stimuli, including interleukin (IL)-1, lipopolysaccharide (LPS) and tumour necrosis factor (TNF) (Gusella et al., 1995; Yeung et al., 1996; Goh et al., 2000), in addition to Type I Interferon (IFN-I), which was regulated by this cytokine and led to its expression (Kuhen and Samuel, 1999; Diebold et al., 2003; Chai et al., 2011). In addition, PKR regulated the activation of nuclear factor (NF)-κB, the key cellular transcriptional component of inflammatory response (Maggi et al., 2000; Zamanian-Daryoush et al., 2000).

Numerous pathogens were able to modulate the signal transduction process that induced or undermined NF-κB activation (Liu et al., 2017). For example, *Leishmania donovani* activated the NF-κB through the mediation of reactive oxygen (Singh et al., 2004). Furthermore, studies showed that purified lipophosphoglycan of *L. mexicana* impaired NF-κB translocation to the nucleus in monocytes, resulting in a subsequent decrease in IL-12 production (Argueta-Donohue *et al.,* 2008). *L. major* amastigotes demonstrated to induce p50/cRel NF-κB complexes, which subsequently resulted in the expression of IL-10 and TNF expression (Guizani-Tabbane et al., 2004). Recent studies have demonstrated that *L. amazonensis* was capable of subverting the p65/p50 NF-κB activation and inducing p50/p50 complexes that inhibited the inducible nitric oxide synthase (iNOS) expression through the PI3K/Akt pathway (Calegari-Silva et al., 2009, 2015). It was also demonstrated that *L. amazonensis*-infected human macrophages, when treated with PolyI:C, were able to modify the NF-κB dimer activated by this PKR inductor and control iNOS (Pereira et al., 2010), with NO production being the main effector mechanism for *Leishmania* elimination by macrophages (Green et al., 1991; Stenger et al., 1994; Wei et al., 1995; Bogdan, 2001; ). An elegant study by Faria and collegues (2014) demonstrated that PKR activation plays an important role in macrophages infected by *Leishmania major*, leading to the parasite death. However, the molecular mechanisms of gene expression regulation have not been addressed until the present work.

In this study, we investigated the function of PKR expression and signalling in the context of *L. major* infection in host macrophages. We examined the necessity of PKR in the expression of NF-κB-dependent inflammatory genes that induced parasite killing. The present study explores the intricate relationship between host macrophage and *L. major* parasites within PKR’s pivotal role, underscoring the importance of these findings in the disease progression.

## Materials and methods

### Cell culture

The human monocytic leukaemia cell line THP-1 (ATCC:TIB202^TM^) and the mouse macrophage leukaemia cell line RAW 264.7 (ATCC: TIB-71) were maintained in high glucose DMEM medium (Vitrocell Embriolife, Campinas, SP, Brazil) supplemented with 10% heat-inactivated fetal bovine serum (Sigma-Aldrich, St. Louis, MO, USA), 100 U/mL penicillin and 100 µg/mL streptomycin in an incubator at 37°C with 5% CO_2_. THP-1 cells were differentiated into macrophages with 40 ng/mL of phorbol-12 myristate-13 acetate (PMA) for 3 days. The cells were then washed thrice with phosphate-buffered saline (PBS) and incubated in fresh medium for another 3 days. RAW 264.7 cells expressing either an empty vector (RAW-Bla cells) or a dominant-negative PKR K296R (RAW-DN-PKR cells) were generated by Silva and colleagues (2004), as previously described. Peritoneal macrophages were induced with thioglycolate from wild-type (WT) or PKR-knockout or IFNR-1^-/-^ 129Sv/Ev mice and obtained by injection of 10 mL DMEM into the peritoneal cavity. The cells were then washed in 1X PBS and resuspended in serum-free DMEM. The cells were plated on 24-well polystyrene plates at a density of 5 × 10^5^ cells per well and incubated at 37°C for 2 h. Non-adherent cells were removed by washing with PBS and the adherent cell population was incubated for 24 h in DMEM containing 10% of fetal bovine serum. The subsequent *Leishmania* infection assays were then performed.

### Luciferase assay

To examine the activity of the PKR promoter, RAW 264.7 cells (1 × 10^5^ cells per well) were placed in 48-well polystyrene plates and transfected using Lipofectamine 2000 reagent (#11668027 ThermoFisher). The p503-WT plasmid (PKR promoter) was kindly provided by Dr. Charles E. Samuel (University of California, Santa Barbara, EUA). The p6kB-Luc plasmid was used to measure NF-kB transcriptional activity (provided by Dr. Patrick Baeuerle). For the measurement of iNOS promoter activity, the plasmids pTK-3XNS or pTK-3XS were provided by Dr. David Geller of University of Pittsburgh, Pennsylvania, EUA. We used 1 μg of all reporter plasmids. To ensure the accuracy of the luciferase readings, 40 ng of the pRL-CMV plasmid (Promega Corp., Madison, WI, USA) was used for normalization. After infection and treatment, the cells were washed with PBS, lysed according to the Dual Luciferase System protocol (Promega) and analysed in the TD-20/20 Luminometer (Turner Designs, Sunnyvale, CA, USA).

### Determination of nitrite

The Griess reaction was used to analyse the nitrite (NO_2_^−^) content as an indicator of NO production in supernatant cultures of RAW 264.7 WT and DN of PKR (2 × 10^5^ cells per well). The standard was then assessed by measuring the absorbance at 540 nm, which was measured after incubation of 50 μL of supernatants with 50 μL of the solution containing *N*-[naphthyl] ethylenediamine dihydrochloride (Need) (1 mg/mL), sulphanilamide (10 mg/mL) and 5% phosphoric acid.

### Immunoblotting

RAW 264.7 cells or peritoneal macrophages (1 × 10^6^ cells) were washed twice with ice-cold PBS (#17-516Q – Lonza) and then lysed in 100 μl of lysis buffer (50 mM Tris-HCl, pH 7.5; 5 mM EDTA; 10 mM EGTA; 50 mM NaF; 20 mM β-glycerophosphate; 250 mM NaCl; 0.1% Triton X-100; and 1 μg/ml BSA) in which a 1:100 dilution of protease inhibitor cocktail (#P8340 – Sigma-Aldrich) was used for total or nuclear protein extraction. The proteins (30 μg) were then subjected to electrophoresis in 10% SDS-polyacrylamide gels and transferred to a nitrocellulose membrane (#GE10600002 – Amersham Biosciences, Piscataway, NJ, USA). The membranes were blocked with 5% nonfat dry milk in TBS with 0.1% of Tween-20 (TBS-T). Subsequently, the blots were subjected to an overnight incubation with (1:500 diluted) antibody against PKR (#3072S), eIF2-α (#9722S), phospho-eIF2-α (#9721S), STAT1α/β (#9172S), phospho-STAT1 (#7649S), GAPDH (#2118S) and Lamin A/C (#2032S) (Cell Signaling Technology, Danvers, MA, USA), NFκB-p50 (#06-886-I) and phospho-PKR Th451 (#07-886) (Millipore, Billerica, MA, USA), NFκB-p65 (#sc-8008) and IkBα (#sc-371) (Santa Cruz Biotechnology, Dallas, Texas, USA), α-tubulin (#T5168) and β-actin (#A2228) (Sigma-Aldrich), followed by anti-rabbit (#A3687 – Sigma-Aldrich) or anti-mouse (#32230 – ThermoFisher) horseradish peroxidase–conjugate IgG (1:2000). The membranes were then subjected to three washings with TBS-T 0.1% following each incubation. Proteins detection was accomplished by the ECL chemiluminescent detection system (#RPN2109 – Amersham Biosciences).

### Parasites, culture conditions and infection

In this study, *Leishmania* (*L.*) *major* (LV-39 MHRO/Sv/59/P) was used. The promastigotes forms were cultivated at 26°C in Schneider’s Insect Medium (#S9895 – Sigma-Aldrich) with 10% fetal bovine serum (#10270106 ThermoFisher), 100 U/mL penicillin and 100 µg/mL streptomycin. Following a period of 4–5 days, the cells from stationary cultures were used for the experimental procedures. The mammalian cells were infected with *Leishmania* promastigotes at a parasite cell ratio of 10:1 for a specified duration at 37°C. In some experiments, THP-1 cells were subjected to treatment with PKRi in conjunction with the infection. The infection index was calculated by multiplying the percentage of infected macrophages by the average number of parasites per infected macrophage, as observed under Giemsa staining.

### Quantitative real-time RT-PCR

The total RNA was extracted from RAW 264.7 WT- and DN-PKR cells (1 × 10^6^ cells) using the Invitrap® Spin Cell RNA mini kit (#1060100200 – STRACTEC Molecular GmbH, Berlin, Germany). Ad 1 µg of the extracted RNA was then reverse-transcribed to first-strand cDNA using ImProm-II (#A3801 – Promega) and oligo(dT) primer, following the manufacturer’s instructions. The DNA sequences of the primers used are described: PKR: F-5ʹ-GATGGAAAATCCCGAACAAGGAG-3ʹ and R-5ʹ-AGGCCCAAAGCAAAGATGTCCAC-3ʹ; IFN-β: F-5ʹ-TCCAAGAAAGGACGAACATTCG-3ʹ and R-5ʹ-TGAGGACATCTCCCACGTCAA-3ʹ; TNF: F-5ʹ-GGTCCCCAAAGGGATGAGAAGTTC-3ʹ and R-5ʹ-CCACTTGGTGGTTTGCTACGACG-3ʹ; iNOS: F-5ʹ-CAGCTGGGCTGTACAAACCTT-3ʹ and R-5ʹ-CATTGGAAGTGAAGCGTTTCG-3ʹ; GAPDH: F-5ʹ-TGCACCACCAACTGCTTAGC-3ʹ and R-5ʹ-GGCATGGACTGTGGTCATGAG-3ʹ. Amplicon specificity was meticulously confirmed by the presence of a single melting temperature peak in dissociation curves executed at real-time reverse transcription polymerase chain reaction (RT-PCR). The experimental quantitative (q)RT-PCR data from the experiments were normalized by way of GAPDH primers as an endogenous control. Through primer validation (different concentrations) and efficiency tests (standard curve – endogenous vs target), the endogenous gene does not vary its expression within the variables of our model, such as infections, times and treatments. The qRT-PCR was conducted using the Applied Biosystems StepOneTM detection system (Applied Biosystems - Massachusetts, USA) with GoTaq® qPCR Master Mix (#A6001 – Promega Corp., Madison, WI, USA). It is noteworthy that all qRT-PCR experiments were performed at least thrice. All expression ratios were calculated using the ΔΔCt method, which is a well-established technique for analysing relative gene expression, with the data analysis being conducted through the StepOne software version 2.0 (Applied Biosystems).

### ChIP assay

Chromatin immunoprecipitation (ChIP) analysis was conducted in accordance with the Simple ChIP Enzymatic Chromatin IP Kit protocol, as outlined in the Cell Signaling (#9004S). RAW 264.7 cells WT- and DN-PKR were plated to confluence in 15-cm dishes. Following infection, the cells were fixed with 1% formaldehyde for a period of 10 min at room temperature. Subsequently, glycine was added to a final concentration of 125 millimoles (mM) for a duration of 5 min at ambient temperature. Subsequently, cell lysis was initiated. Subsequently, one unit of Micrococcal Nuclease was added to the sample and incubated for 20 min at 37°C to digest DNA to the length of approximately 150–800 bp. Subsequently, the chromatin was immunoprecipitated with 2 µg/mL of anti-STAT1, anti-p50 or anti-p65 antibodies at 4°C under rotation for 16 h. The DNA isolated from the immunoprecipitated material was amplified by real-time PCR using SYBR Green and the forward and reverse primers, respectively: PKR promoter: F-5ʹ-GGGTACAGAGGCGACACGCCTA-3ʹ and R-5ʹ-TTCCCTGCCGCTGCTGCT-3ʹ; TNF-κB#3-F-5ʹ-GCCCTCCCAAAGCCCATGC-3ʹ and R-5ʹ-GCATGGGGGGGTGCTTCTGA-3ʹ; TNF-κB#1-F-5ʹ-GTGACTCCACTGGAGGGTGGGAG-3ʹ and R-5ʹ-CCCACAGCCCTGCTTCCAGG-3ʹ; IFN-β promoter: F-5ʹCCCAGTACATAGCATATAAAATACCA-3ʹ and R-5ʹ- GGGATGGTCCTTTCTGCCT-3ʹ. To establish a control, 1/50 of the digested input chromatin was subjected to a parallel processing and analysis strategy in the absence of immunoprecipitation. The input percentage of the samples was calculated by adjusting the input to 100% (average Ct of input – Log2 of 50) followed by the application of the 100 × 2^(adjusted input – average Ct(IP))^ formula.

### Reagents and antibodies

PMA (#19-144), polyinosinic:polycytidylic acid [PolyI:C] (#P1530), LPS (#L2630), wedelolactone (#W4016) and BAY11-7082 (#B5556) were procured from Sigma-Aldrich (St. Louis, MO, USA). A PKR inhibitor (#527450) was procured from Millipore (Darmstadt, Germany), and human recombinant interferon-alpha 2b was obtained from Blausiegel (Cotia, SP, Brazil).

### Statistical analysis

Statistical analyses were conducted using Prism 7.0 (GraphPad Software) with Student’s *t*-test and two-way analysis of variance (ANOVA). A *p*-value less than 0.05 was considered statistically significant. The data were analysed as described in the figure legends, and the number of technical or biological replicates used to generate the graphs, as well as the number of independent assays or experiments performed and yielding similar trends between groups, were also given in the figure captions. In light of the observed variation in absolute numbers across independent assays involving peritoneal thioglycolate-recruited macrophages, which is potentially attributable to differing activation states at the time of collection, a statistical analysis was conducted. This analysis employed technical replicates for each group within each independent assay.

## Results

### L. major shows a reduction in the infection index due to the activation of PKR/IFN-I signalling

**Figure 1.**
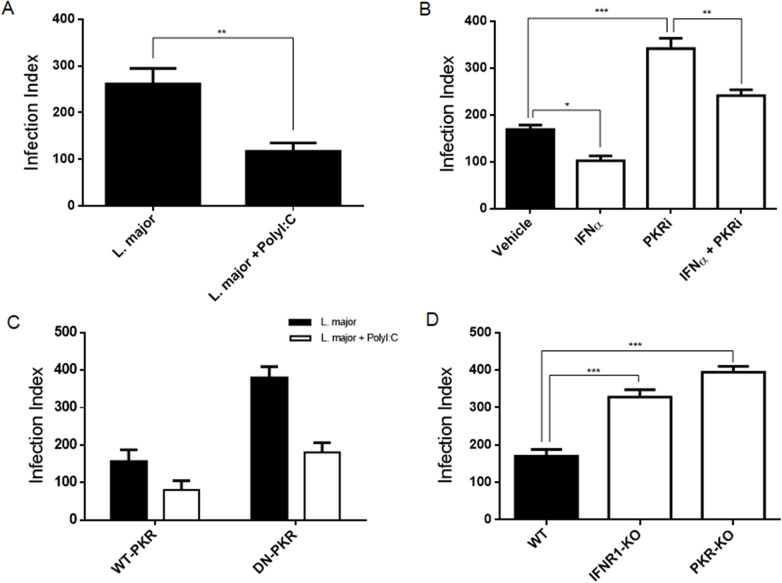
The intracellular proliferation of *L. major* is influenced by the activation of the PKR/IFN-I axis. THP-1 cells were infected with stationary promastigotes forms of *L. major* for 24 h. Then they were treated with PolyI:C for an additional 24 h (A). The same cells were infected for 24 h and, after this time, and then the PKR inhibitor (iPKR) and/or recombinant interferon alpha were added for an additional 24 h (B). After this time, the cells were fixed, and the infection index was evaluated. (C) RAW-WT-PKR and RAW-DN-PKR cells were infected with stationary promastigotes forms of *L. major* for 24 h. Then, they were treated with PolyI:C for an additional 24 h. During this time, the number of parasites inside the cells was counted, and the infection index was calculated. (D) Peritoneal macrophages from wild-type, PKR-ko or IFNR1^-/-^ 129/sv mice were infected with stationary promastigotes forms of *L. major* for 48 h. After this time, the cells were fixed, and the infection index was evaluated. Statistical analysis was carried out using the Student’s *t*-test. The results were representative of three independent experiments. *P < 0.05, **P < 0.01, ***P < 0.001.

The primary objective of this study was to investigate whether *L. major* exerts analogous effects to *L. amazonensis* in requiring PKR signalling increased parasite proliferation in macrophages. The initial findings suggested that PolyI:C treatment, a well-established PKR inducer, resulted in a reduction in infection levels in THP-1 cells infected with *L. major* (Figure 1A). As demonstrated in Figure 1B, the treatment with recombinant IFN-α, a PKR inducer, was opposed to treatment with PKRi relative to the control. In order to confirm the role of the PKR/IFN axis in *L. major* infection, RAW 264.7 cells with a deleted PKR (DN-PKR) or with a WT-PKR were infected and treated with PolyI:C 24 h later to analyse parasite load (Figure 1C). In DN-PKR cells, *L. major* proliferation was favoured, indicating the requirement of PKR signalling to impair *L. major* proliferation. To utilize the model in which PKR or IFN-I signalling were knockout, we infected PKR^-/-^ and IFN-R1^-/-^ peritoneal macrophages. The measurement showed that the spread of infection is increased by knockout cells (Figure 1D). The results of the study demonstrated that PKR/IFN-I-deficient cells promote an increase in *L. major* proliferation, and that the action of this kinase and cytokine possibly denotes a positive aspect in controlling the infection.

### L. major *induces phosphorylation of PKR and inhibits its expression*

**Figure 2.**
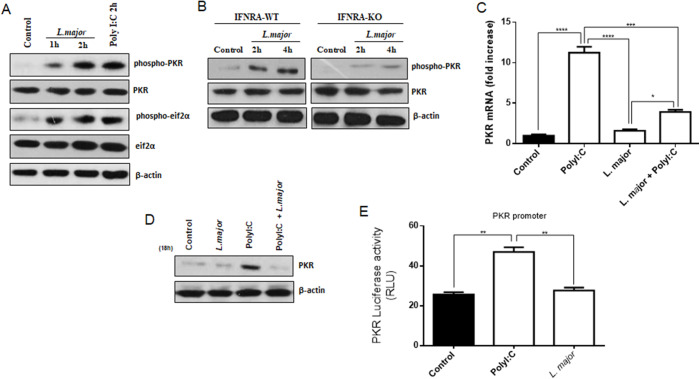
*L. major* infection can modulate PKR activity and expression. RAW 264.7 (A) and peritoneal macrophages from wild-type or IFNR1^-/-^ (B) were infected with stationary promastigotes forms of *L. major* at indicated times. Western blot was used to extract the total protein with anti-phospho-PKR, total PKR, anti-phospho-eIF2α or total eIF2α antibodies. (C) Cells were infected with stationary promastigotes of *L. Major* for 4 h, or together with PolyI:C, or PolyI:C alone, for additional 1 h post infection, were harvested, and total RNA was extracted. Then, a quantitative real-time RT-PCR assayed was performed. (D) RAW 264.7 cells were infected with stationary promastigote forms of *L. Major* or treated with PolyI:C for 18 h and Western blot was carried out for total protein extract with anti-PKR. (E) RAW 264.7 cells were transiently transfected using a reporter plasmid p503-WT that contains KCS and ISRE elements upstream of the luciferase reporter gene. 24 h later, post-transfection cells were infected with stationary promastigotes forms of *L. Major* or treated with PolyI:C. After 24 h, the whole-cell lysates were checked for luciferase activity. Statistical analysis was carried out using the Student’s *t*-test. The results are from three independent experiments. *P < 0.05, **P < 0.01, ***P < 0.001, ****P < 0.0001.

In accordance with the recent findings indicating a decline in *L. major* proliferation due to PKR/IFN-I signalling in host macrophages, the phosphorylation levels of PKR were quantified, along with its primary target, eIF2α, within the context of the infection. To analyse PKR activation, Western-blot assays were performed in RAW 264.7 and peritoneal macrophages infected by *L. major* for 2 and 4 h. The results obtained from these experiments demonstrated that the phosphorylation of PKR decreased in RAW 264.7 and IFNR1^-/-^ macrophages following infection by *L. major* promastigotes (see Figure 2A and 2B). The results of this study indicate that *L. major* infection led to PKR activation in an IFN-I-dependent manner. Subsequently, an investigation was conducted to ascertain whether *L. major* infection also increased PKR levels. However, the findings revealed that *L. major* infection did not induce an increase of PKR expression, in addition to being able to reduce the expression increased by PolyI:C treatment. This suggests that there is an inhibitory regulation by the parasite on PKR expression (Figure 2C and 2D). The analysis through gene-reporter assay analysis revealed that the activation of PKR promoter was not induced by *L. major* infection through an unknown mechanism (Figure 2E). These findings suggest that *L. major* employed a mechanism that impeded the induction of PKR gene expression for its own benefit during the establishment of infection in the host cell.

### L. major inhibits STAT1-dependent PKR expression and PKR-dependent STAT1 activation

**Figure 3.**
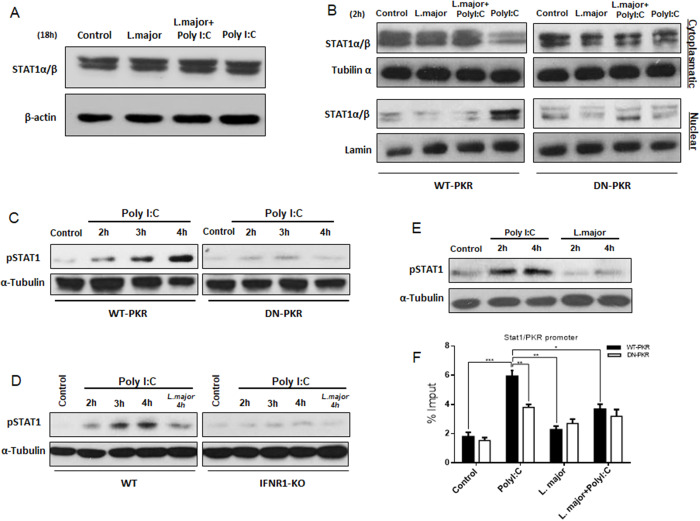
The transcriptional factor STAT1 is inhibited by *L. major* in macrophages. (A) Peritoneal macrophages from wild-type C57BL/6 mice were infected with stationary promastigote forms of *L. Major*, treated with PolyI:C, or infected and treated together with PolyI:C for 18 h. Then, Western-blot analysis was performed on total protein extracts with anti-STAT1α/β. (B) RAW 264.7WT or DN-PKR cells were infected with stationary promastigotes forms of *L. Major* and/or treated with PolyI:C for 2 h. The cytoplasmatic and nuclear proteins were extracted and Western blot was performed with anti-STAT1α/β. (C)–(E) RAW 264.7 WT or DN-PKR cells, peritoneal macrophages from wild-type or IFNR1^-/-^ 129/sv mice and peritoneal macrophages from wild-type C57BL/6 mice, respectively, were infected with stationary promastigotes forms of *L. major* at the indicated time. Western blot was carried out for total protein extract with phospho-STAT1 antibody. (F) RAW 264.7 WT or DN-PKR cells were infected with stationary promastigotes forms of *L. major* for 4 h, or together with PolyI:C for additional 1 h, and then submitted for chromatin immunoprecipitation assay (ChIP) using STAT1 ChIP-antibody. Statistical analysis was carried out using the two-way ANOVA method. The results are representative of three independent experiments. **P < 0.01, ***P < 0.001.

The activation of STAT1 was described in the context of host resistance to intracellular infection by *L. major* (Späth et al., 2009), despite the observation that *L. mexicana* and *L. major* decreased the STAT1 phosphorylation (Bhardwaj et al., 2005). The initial inquiry focused on whether *L. major* inhibited PKR levels by hindering the translocation of STAT1 to the nucleus and its binding to the PKR promoter. In order to investigate the hypothesis that *L. major* decreased STAT1 levels, Western-blot assays were performed in cells infected and treated with PolyI:C, either in combination or separately. The results demonstrated that total levels of STAT1 remained constant after 18 h of infection/treatment (Figure 3A). A more detailed analysis was then performed in cytoplasmic and nuclear extracts, where it was verified that *L. major* inhibited STAT1 translocation to nuclei induced by PolyI:C in a PKR-dependent manner (Figure 3B). As observed, the parasite manages to reduce the nuclear translocation of STAT1, which is stimulated by PolyI:C treatment. Moreover, an augmentation in STAT1 phosphorylation was detected, induced by PolyI:C in a PKR/IFN-I-dependent manner, in the absence of infection (see Figure 3C and 3D). In addition to impeding the translocation of STAT1 to the nucleus, the *L. major* infection did not induce STAT1 phosphorylation (Figure 3E). Consequently, this impeded the binding of the transcription factor to the PKR promoter, as analysed by the ChIP experiment at the ISRE-binding site on the mouse PKR promoter in a *L. major* infection and PolyI:C treatment dependent manner (Figure 3F). These findings support the observation that in *L. major* infection, there was an inhibition of PKR expression due to the inhibition of STAT1 translocation and phosphorylation. This, in turn, prevented the augmentation of downstream signals that were disadvantageous to the parasite.

### PK-mediated NF-κB activation is induced by L. major infection

**Figure 4.**
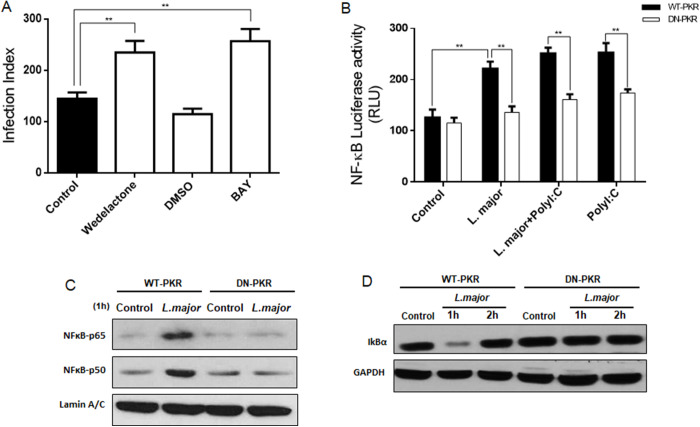
Infection activates NF-κB in a PKR-dependent manner. (A) THP-1 cells were differentiated with PMA and then treated with wedelolactone and BAY for 2 h previously infection with stationary promastigotes of *L. major* for 48 h. After this time, the cells were fixed, and the infection index was evaluated. (B) RAW 264.7WT-PKR and DN-PKR cells were transiently transfected with NF-κb luciferase reporter construction (6κB-Luc). And 24 h after the cells were exposed to the transfection, they were infected with stationary promastigote forms of *L. Major* or treated with PolyI:C. After 24 h, the whole-cell lysates were analysed for luciferase activity. RAW 264.7 WT-PKR and DN-PKR cells were infected with stationary promastigote forms of *L. Major* at the indicated time. Nuclear (C) or total (D) protein extracts were obtained. Western blot was carried out with anti-p65, anti-p50 and anti-iκBα, respectively. Statistical analysis was carried out using the two-way ANOVA method. The results are representative of three independent experiments. **P < 0.01.

Recent findings have demonstrated that *L. amazonensis* activates the p50/p50 NF-κB, a well-established transcriptional repressor complex, in both murine and human macrophages (Calegari-Silva et al., 2018). Furthermore, the treatment with PolyI:C demonstrated a synergistic enhancement of the p50/p50 homodimer (Pereira et al., 2010). In contrast, *L. major* induced the rp65/p50 complex, a classical transcriptional activator (Calegari-Silva et al., 2009). In addition, an increase in the activation of this heterodimer was observed following PolyI:C treatment. To further characterize the ability of *L. major* to induce downstream effectors in PKR signalling, PKR-dependent NF-κB activation was also investigated. It is established that NF-κB modulates a number of pro-inflammatory genes; consequently, the primary objective of this study was to ascertain whether the suppression of this transcription would impact the proliferation of *Leishmania*. As demonstrated in Figure 4A, the parasites exhibited enhanced proliferation when NF-κB activation was inhibited through the administration of wedelolactone and BAY11-7082, two NF-κB inhibitors. Subsequently, RAW 264.7 WT-PKR and DN-PKR were transiently transfected with NF-κB luciferase reporter construction (6κB-Luc). The following day, the cells were infected with Leishmania and/or treated with PolyI:C for an additional 24 h prior to luciferase assay. The results demonstrated that PolyI:C induced the activation of NF-κB consensus Luciferase promoter, and *L. major* infection displayed the same results in a PKR-dependent manner (Figure 4B). In order to provide further substantiation for these observations, nuclear extracts from WT- or DN-PKR RAW 264.7 cells were subjected to Western-blot analysis. The membrane was then incubated with α-p50 and α-p65 antibodies to assess PKR-dependent NF-κB nuclear translocation. As anticipated, a decrease in the nuclear translocation of p65 and p50 was evident in DN-PKR cells following *L. major* infection (Figure 4C). In addition to these results, the decrease of IκBα was only observed in PKR-WT cells (Figure 4D), indicating that PKR-dependent activation of NF-κB was induced by *L. major* and possibly the inflammatory genes regulated by this transcription factor are downregulated in the absence of PKR signalling.

### PK/NF-κB-dependent iNOS expression and NO production by L. major infection

**Figure 5.**
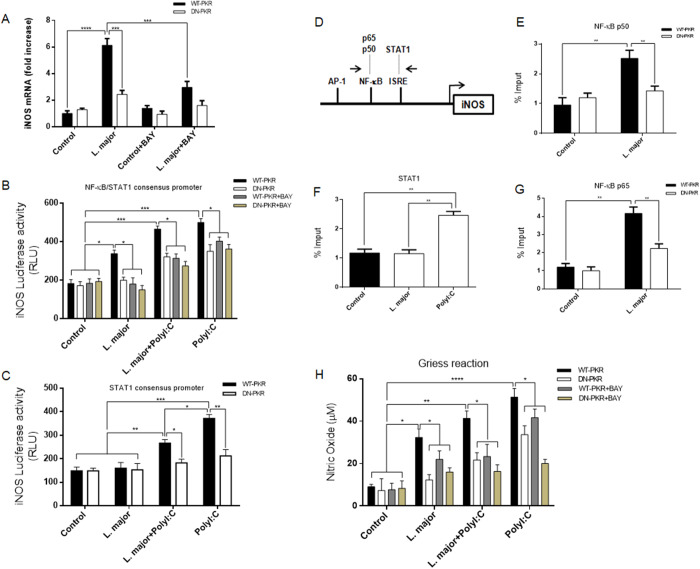
Nitric oxide synthesis and iNOS expression by *L. major* infection in a PKR/NF-κB-dependent manner. (A) RAW 264.7WT-PKR and DN-PKR cells were infected with stationary promastigote forms of *L. major* for 4 h or treated with BAY. The cells were harvested, and total RNA was extracted and then analysed by a quantitative real-time PCR using murine iNOS primers. The same cells were transiently transfected with pTK-3XNS (B) or pTK-3XS (C) luciferase plasmids. And 24 h after the cells were exposed to the transfection, they were infected with stationary promastigote forms of *L. Major* or treated with PolyI:C and BAY. After 24 h, the whole cells were checked for luciferase activity. RAW 264.7WT or DN-PKR cells were infected with stationary promastigote forms of *L. Major* for 4 h. Then, they were submitted for a special kind of DNA test called a ‘chromatin immunoprecipitation assay (ChIP)’ in the iNOS promoter (D) using p50 (E), STAT1 (F) and p65 (G) ChIP-antibodies. RAW 264.7 WT-PKR and DN-PKR cells were infected with *L. major* and/or treated with PolyI:C. Twenty-four hours later, the supernatants were collected and the nitrite concentrations evaluated by Griess reaction (H). Statistical analysis was carried out using the two-way ANOVA method. The results are representative of three independent experiments. *P < 0.05, **P < 0.01, ***P < 0.001, ****P < 0.0001.

It was demonstrated that *L. amazonensis* inhibited the production of nitric oxide (NO) in macrophages. This inhibition was induced by p50/p50 NF-κB-repression of iNOS promoter (Calegari-Silva et al., 2015). In addition, *L. amazonensis* was able to inhibit NO increase levels induced by PolyI:C, subverting p65/p50 NF-κB heterodimer, in a PKR-dependent manner (Pereira et al., 2010). An analysis of iNOS transcripts levels in WT-PKR or DN-PKR cells was conducted to demonstrate that these effects are distinct in *L. major* infection in a PKR-dependent manner. *L. major* infection induced sixfold increase in iNOS expression in WT-PKR cells. The results revealed that *L. major* infection elicited a sixfold augmentation in iNOS expression in WT-PKR cells, while it was only partially induced in DN-PKR cells or in cells treated with BAY11-7082 (Figure 5A). Subsequently, a Luciferase reporter assay was performed to characterize the modulation on iNOS promoter. To this end, RAW 264.7WT-PKR or DN-PKR cells were transiently infected with a pTK-3XNS plasmid, which contained three copies of NF-κB/STAT1 motifs corresponding to −5.8 kb of the iNOS promoter. A second pTK-3X plasmid was similarly utilized, corresponding to the sole STAT1 motifs at −5.2 kb of the iNOS promoter. The following day, the cells were infected with *L. major* and then treated with PolyI:C or BAY11-7082. Following a 24-h period, the luciferase activity was evaluated. As demonstrated in Figure 5B, *L. major* instigated the activation of iNOS promoter in a PKR/NF-κB-dependent manner. This observation was further confirmed by the finding that *L. major* did not induce STAT1 transcriptional activity on the iNOS promoter (Figure 5C). In a ChIP assay, it was confirmed that both p65 and p50 binded to the iNOS promoter (Figure 5D) in a PKR-dependent manner, unlikely STAT1 (Figure 5E–5G). To validate these observations, PolyI:C treatment resulted in a substantial augmentation of NO production in WT-PKR cells, as evidenced by Griess reaction (Figure 5H). The results of this study indicated that PKR/NF-κB-dependent NO production by *L. major* partially contributed to the increase in proliferation observed in PKR-absent cells. These findings provide substantial evidence that iNOS plays a regulatory role in *L. major* infection of macrophages, a function that is contingent upon PKR signalling.

### PK signalling is required for NF-κB-dependent IFN-β expression in L. major infection

**Figure 6.**
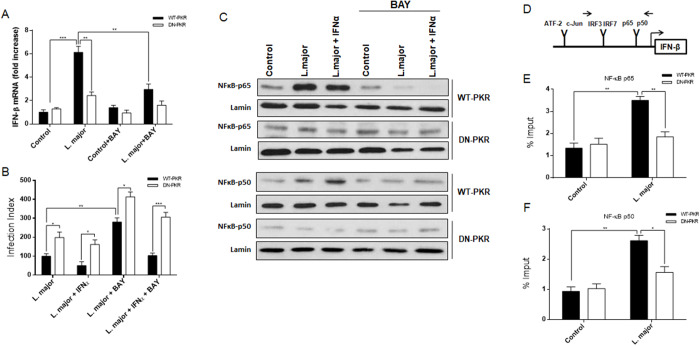
*L. major* increases interferon-β expression by activating the PKR/NF-κB axis. All assays were performed on RAW 264.7WT-PKR and DN-PKR cells. (A) Macrophages were infected with stationary promastigote forms of *L. major* for 4 h or treated with BAY and total RNA was extracted followed by a quantitative real-time RT-PCR was assayed. (B) The same cells were previously treated with recombinant IFNα or BAY and then infected with *L. Major* for 48 h. After this time, the cells were fixed, and the infection index was evaluated. (C) Nuclear protein extracts of infected or BAY treatment cells were analysed using p65 and p50 antibodies. The interferon-β promoter (D) of macrophages were infected with stationary promastigotes forms of *L. major* for 4 h and then submitted for ChIP assay using p65 (E) and p50 (F) ChIP-antibodies. Statistical analysis was carried out using the two-way ANOVA method. The results are representative of three independent experiments. *P < 0.05, **P < 0.01, ***P < 0.001.

It is well established that PKR modulated IFN-I production (Schulz et al., 2010), and that this cytokine performed a multifaceted role in the progression of *Leishmania* infection on macrophages (Shankar et al., 1996; Diefenbach et al., 1998). Previous studies demonstrated that *L. amazonensis* induced IFN-β production in a PKR-dependent manner via TLR2 engagement (Vivarini et al., 2011). An analysis of IFN-β transcripts expression in infected host macrophages was conducted to elucidate the primary mechanism that promoted the observed discrepancy in PKR-dependent Leishmania infection index. As with iNOS expression, infection by *L. major* induced IFN-β expression in a PKR/NF-κB-dependent manner showing a marked reduction in infected macrophages expressing DN-PKR or treated with BAY11-7082 (Figure 6A). The biological relevance of this observation was confirmed in RAW 264.7 cells treated with recombinant IFN-α. A notable finding was the observation that rec-IFN-α exhibited a tendency to reduce the parasite load associated with *L. major* infection (Figure 6B), thereby suggesting a discrepancy in the outcomes of the infection. This finding serves to reinforce the established role of PKR in the production of IFN-I by either parasite. Furthermore, the analysis of nuclear translocation of the subunits p65 and p50 indicated that when treated with IFN-α and/or BAY11-7082, the dependency of PKR signalling in mediating IFN-α-induced NF-κB activation was strengthened (Figure 6C). To verify the PKR/NF-kB-dependent IFN-β expression, the ChIP analysis revealed that both p65 and p50 enhanced binding in the IFN-β promoter (Figure 6D) during *L. major* infection, exclusively in WT-PKR cells (Figure 6E and 6F). The present findings indicate that *L. major* augmented IFN-β expression in a PKR-dependent manner, thereby inducing NF-kB signaling and establishing a positive regulatory loop within the system.

### L. major induces TNF expression via PKR/ NF-κB pathway

**Figure 7.**
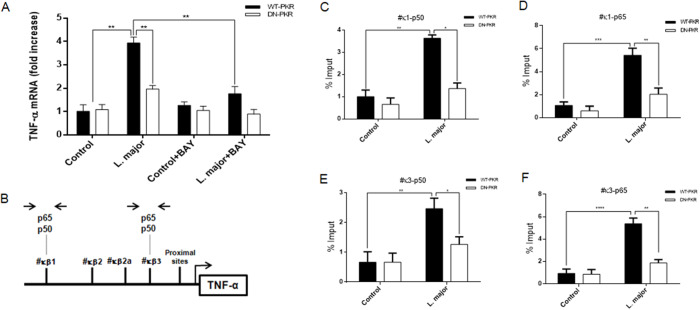
TNF expression due to parasite infection depends on PKR/NF-κB signalling. All tests were done on RAW 264.7WT-PKR and DN-PKR cells. (A) Macrophages were infected with stationary promastigote forms of *L. major* for 4 h or treated with BAY. Total RNA was extracted, and a quantitative real-time RT-PCR assayed was performed to measure TNF transcripts. Macrophages were infected with stationary promastigote forms of *L. major* for 4 h, then, TNF promoter (B) occupancy was assessed. The extracted chromatin was submitted for ChIP assay using p50 and p65 (C, D) to the #κβ1 site, and the same antibodies to the #κβ3 site (E, F), respectively. Statistical analysis was carried out using the two-way ANOVA method. The results are representative of three independent experiments. *P < 0.05, **P < 0.01, ***P < 0.001.

The PKR has also been implicated in the control of TNF expression, regulating the splicing of mRNA precursor transcripts, consequently increasing a positive feedback loop due to a TNF-stimulated PKR activation (Osman et al., 1999). TNF has been described as modulating Leishmania infection for some time (Liew et al., 1990). In order to conduct a more thorough investigation into whether an infection by *L. major* results in PKR/NF-kB-dependent TNF expression, RAW 264.7 cells were subjected to infection for a period of 4 h. Subsequently, a qRT-PCR assay was performed on the total RNA. The results demonstrated that *L. major* was capable of inducing TNF expression in a PKR/NF-kB-dependent manner (Figure 7A). Subsequent analyses were conducted using a ChIP assay. These analyses confirmed the enhanced occupancy of both p50 and p65 transcription factors on the #β1 and #β3 promoter sites within the TNF gene (Figure 7B), and this occurred in a PKR-dependent manner (Figure 7C to 7F). These results suggest that PKR activated the NF-κB transcription factor in *L. major* infection, which in turn increased TNF levels, leading to a decrease in parasite load.

## Discussion

The interaction of parasites with mediators and components of innate immunity represents a pivotal step in the maintenance of equilibrium between resistance and host susceptibility to leishmaniasis (Mota et al., 2021). The activation of PKR in several signalling pathways has been shown to culminate in the modulation of a gene expression profile during major infectious processes (García et al., 2006; Balachandran and Barber, 2007). As previously reported, the role of PKR in the macrophage response to *L. amazonensis* was documented (Pereira et al., 2010; Vivarini et al., 2011, 2015, 2017). This observation revealed a different sophisticated evolutionary mechanism involving *L. major* infection. This mechanism proved incapable of counteracting the PKR-dependent NF-κB activation induced by PolyI:C treatment. Consequently, the production of mediators was initiated, leading to the destruction of the intracellular parasites.

Initial observations indicated that *L. major* infection was disfavoured in PolyI:C-activated macrophage cells in a PKR and IFN-dependent manner. In addition to these results, it was verified that *L. major* induces the phosphorylation of the PKR protein, and also a decrease in PKR phosphorylation in the absence of the IFN-I receptor. These findings suggest that *L. major* infection does not inhibit PKR activation, thereby maintaining the integrity of the downstream signals that regulate the host cell’s immune response. The results demonstrated that the activation of PKR by *L. major* was crucial for the microbicidal action of macrophages. A positive feedback loop was identified between PKR activation in IFN-I expression and, consequently, the autocrine loop that regulated PKR gene expression (Pindel and Sadler, 2011). The central question guiding this study is whether *L. major* has the capacity to stimulate PKR gene expression, given the established evidence of IFN-I production during infection (Shankar et al., 1996). To assess this process, it was observed that *L. major* was able to inhibit PKR transcripts, protein levels and promoter activation. The inhibition of PKR expression by *L. major* may be a protective mechanism of this parasite, since this kinase is able to induce the expression of mediators unfavourable to increase *L. major* infection.

The STAT1 transcription factor is a cytoplasmatic protein which plays a critical role in signalling of several cytokines (Boehm et al., 1997). Numerous studies indicated that *L. mexicana, L. donovani* and *L. major* were able to inhibit STAT1 signalling in macrophages (Bhardwaj et al., 2005; Späth et al., 2009; Matte and Descoteaux, 2010). Furthermore, STAT1 was required in PKR promoter activation in a trimeric complex, named ISGF3 (STAT1, STAT2 and IRF9) (Kuhen and Samuel, 1999). Subsequent observation indicated that following PolyI:C treatment, infection with *L. major* resulted in the inhibition of nuclear translocation of STAT1 in a PKR-dependent manner and consequently affected the PKR promoter occupancy. Furthermore, the parasites have been observed to impede the activation of STAT1 by phosphorylation, a process that is also PKR-dependent. This observation pertains to the downstream signalling. The activation of PKR by PolyI:C has been shown to result in increased STAT1 phosphorylation, a process that is dependent on this kinase (Ruuska et al., 2012). This, in turn, has been demonstrated to promote positive feedback on the expression of PKR itself. It is noteworthy that PKR does not directly phosphorylate STAT1, as this interaction is independent of its catalytic activity (Wong et al., 1997). Thus, it can be concluded that *L. major* inhibited the PKR gene expression at the promoter level by downregulating the STAT1 activation by mechanisms still to be investigated.

PKR is a central kinase that regulates numerous inflammatory genes, some of which are NF-kB dependent (Bonnet et al., 2000; Gil et al., 2000; Kang and Tang, 2012). Infections with *L. major* in c-Rel knockout mice showed an increase in susceptibility to infection (Grigoriadis et al., 1996). NF-kB was also activated in dendritic cells infected by *L. major.* This activation, in conjunction with the IRF1 and IRF8, resulted in an increase in the inflammatory cytokine IL-12 (Jayakumar et al., 2008). The data demonstrated that NF-κB activation, induced by infection, occurred exclusively in cells containing PKR, as evidenced by the nuclear translocation of the p65 and p50 subunits. This observation corroborates the previously reported data concerning p65 nuclear localization (Faria et al., 2014). The modulation of consensus binding sites of NF-κB was contingent upon PKR signalling and the different dimmers of NF-κB induced by *L. major* infection. These data demonstrate that the main mechanism regulating NF-κB was dependent of PKR signalling. These results corroborate previous findings in the literature, particularly regarding the interaction between PKR and the primary components of the NF-kB pathway (Bonnet et al., 2000, 2006).

Elevated levels of NO, which are produced by the action of the enzyme iNOS and are regulated by PKR, is one of the main strategies for the elimination of intracellular pathogens in host cells, such as *L. tropica* and *L. mexicana* (Qadoumi et al., 2002; Zafra et al., 2008; Kumar et al., 2010). In contrast, the infection by *L. amazonensis* inhibited iNOS induction by subverting NF-κB signalling that bind in iNOS promoter (Calegari-Silva et al., 2009). The present study demonstrated that *L. major* induced PKR/NFKB-dependent iNOS expression and NO secretion in supernatant. The expression of iNOS in PKR DN cells had previously been analysed (Faria et al., 2014), but this expression was not associated with dependence on the PKR/NF-kB axis. These results indicated that in the absence of PKR/NF-kB signalling, iNOS expression was reduced, thereby favouring *L. major* proliferation in host cells.

In addition to that, several parasitic protozoa modulated the IFN-I signalling in host cells (Bogdan et al., 2004). For instance, *L. major* itself was capable of inducing the expression of IFN-α/β in both i*n vivo* and *in vitro* settings, leading to the expression of iNOS and subsequent production of NO by macrophages through activation (Mattner et al., (2000); Diefenbach et al., 1998). It was demonstrated that *L. major* induced PKR/NFKB-dependent IFN-β gene expression and decrease in parasite growth when recombinant IFN-α was added to infected culture cells. It showed that the PKR regulated the NFKB activation, and that this transcription factor upregulated the IFNβ expression ins a proximal promoter gene by ChIP assay. These results indicate that PKR activation may contribute to an inflammatory profile and, consequently, a high parasite burden.

PKR demonstrated to play a pivotal role in the stabilization of TNF signalling, thereby facilitating the activation of NF-κB (Kumar et al., 1997; Cheshire et al., 1999). In addition, PKR showed to enhance the activation of NF-κB by IFN-γ in conjunction with TNF. The proximal sites at the human or mouse TNF promoter were composed of AP1, ETs, ERG1 and IRS transcription factors. However, transcriptional regulatory activity was enhanced by distal κB sites induced by LPS treatment (Kuprash et al., 1999). In mice lacking TNF, the inflammatory macrophages exhibited reduced NO release, and *L. major* rapidly induced host death (Bogdan, 2001; Wilhelm et al., 2001). The results of these studies demonstrated that *L. major* increased TNF transcript in a PKR/NF-κB-dependent manner and that the parasite induced the binding of p65/p50 NF-κB complex in the TNF promoter. Together with iNOS and IFN-β, the expression of TNF in host macrophages allowed the elimination of intracellular parasites by the host macrophage.

**Figure 8.**
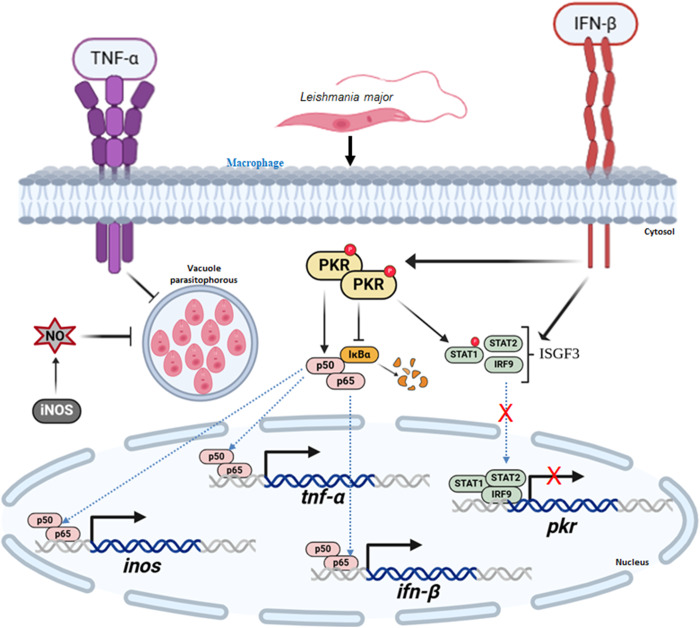
Proposed model comprised by PKR-NF-κB axis in *L. major* infection. When parasites are inside the cell, they trigger a process that leads to the activation of NF-kB, along with the inhibition of iκBα. The process of phosphorylation of STAT1 also depends on PKR. These transcription factors move from the cytoplasm to the nucleus, where they find sites on the genes that control the parasite’s growth or removal. In a certain way, the PKR and NF-κB increase the gene expression of IFNβ, iNOS and TNF. However, they do not increase the PKR gene. This is because STAT1 is not occupied, and it would be complexed with STAT2 + IRF9, generating ISGF3. This is happening even though it is stimulated by the binding of secreted IFNβ to IFNR1. The PKR/NF-kB cascade leads to the production of nitric oxide by increasing the expression of iNOS. This oxidative stress is one of the factors that reduce the intracellular proliferation of *L. major.*

The elucidation of the modulation of intracellular signalling pathways opens alternative proposals on probable interventions aimed at maintaining the system that becomes imbalanced due to infections and external factors. While the host cell may be partially prepared to intervene in opportunistic infections that break homeostasis, parasites, irrespective of their phylogenetic origin, attempt to subvert these pathways on the host. However, the manner in which the host cell responded to diverse pathogens exhibited significant variation. This variation was a critical factor that determined the progression of the disease. Consequently, elucidating the mechanisms by which the innate immune system detects and responds to protozoan parasites is imperative to comprehend the methods through which infection can be controlled. Due to its strong pleiotropic effects and its essential function in normal homeostasis, PKR itself is not yet a target of choice for therapeutic intervention in Localized Cutaneous Leishmaniasis (LCL). Nevertheless, this study corroborates the previous suggestion that downstream targets of the PKR/NF-kB pathway constitute optimal therapeutic targets in LCL. The schematic model, which was developed based on the results, is shown in Figure 8.
